# Is it in their words? Teachers' enthusiasm and their natural language in class–A sentiment analysis approach

**DOI:** 10.1111/bjep.12734

**Published:** 2025-01-31

**Authors:** Anne C. Frenzel, Hannah Kleen, Anton K. G. Marx, David F. Sachs, Franziska Baier‐Mosch, Mareike Kunter

**Affiliations:** ^1^ Department of Psychology Ludwig‐Maximilians‐Universität München Munich Germany; ^2^ DIPF|Leibniz Institute for Research and Information in Education Frankfurt Germany; ^3^ University Siegen Siegen Germany; ^4^ Goethe University Frankfurt Frankfurt Germany

**Keywords:** natural language processing, self‐report, sentiment analysis, teacher enthusiasm

## Abstract

**Introduction:**

Teacher enthusiasm is an undisputedly important characteristic of teachers, with demonstrated positive effects on student outcomes. Existing research typically operationalised teacher enthusiasm via trait‐based teacher‐ or student ratings. Strikingly little is known about how teachers' trait enthusiasm manifests in their actual in‐situ classroom behaviour. Some findings have been reported regarding teachers' nonverbal behaviours, but the links between teacher enthusiasm and teacher language are unknown so far.

**Methods:**

The present contribution fills this research gap by applying lexicon‐based sentiment analysis to quantify teachers' emotional tone from transcribed teacher utterances obtained from video recordings of full mathematics lessons (45 min). *N* = 19 secondary school mathematics teachers and their *N* = 393 students participated in our study. We realised the sentiment analysis using Remus et al.'s emotion lexicon SentiWS (v2.0, 2019). We obtained both teacher self‐reports and student ratings to assess teachers' enthusiasm, shown habitually (trait), and during the videotaped lesson (in situ).

**Results:**

Regarding trait enthusiasm, teachers' own, but not the students', ratings were positively linked with teachers' verbally expressed sentiment in the videotaped lesson, specifically its positive valence. Regarding in‐situ enthusiasm, associations were even larger but also did not reach significance for the student ratings.

**Conclusion:**

This is the first study to employ sentiment analysis on transcripts of German teachers' in‐class talk. Besides the quantitative links between teacher enthusiasm and their language, it also provides qualitative insights on positive emotional teacher talk in mathematics.

## INTRODUCTION

Teachers' enthusiasm has been empirically shown to be positively linked with students' motivation and performance (Bieg et al., 2022; Burić & Frenzel, 2019; Keller et al., 2016; Moè et al., 2021). There is compelling evidence that highly enthusiastic teachers provide a supportive classroom climate and motivating teaching style (Fauth et al., 2019; Lazarides et al., 2021; Moè & Katz, 2022). Conceptually, a differentiation between authentic and inauthentic enthusiasm has been proposed (Keller et al., 2018; Taxer & Frenzel, 2018). In the present study, we address authentic enthusiasm as teachers' internally experienced positive emotion during teaching, coupled with outward positive expressiveness (Keller et al., 2016). Further, habitual and more situated accounts of teacher enthusiasm can be differentiated. Most existing research conceptualised enthusiasm as a personality characteristic, thus assuming that teachers differ from one another in their typical, habitual levels of enthusiasm (Keller et al., 2016). However, it is also conceivable that a teacher's in‐situ enthusiasm varies from lesson to lesson (e.g., Keller et al., 2018). The present study considers both habitual and in‐situ enthusiasm.

Despite a substantive body of empirical evidence on the proposed nature of teacher enthusiasm and its positive effects on students, to date, little is known about what it is that sets highly enthusiastic teachers apart from less enthusiastic teachers, and theoretically well‐founded explanations and empirical evidence on the specific behavioural manifestations of teacher enthusiasm are rare (Keller et al., 2016). In the present contribution, we investigate one specific potential mechanism not yet considered in teacher enthusiasm research, namely the sentiment of the teachers' language. We hypothesise that highly enthusiastic teachers speak differently than their less enthusiastic colleagues in that they use more verbal expressions with a positive emotional connotation in the classroom. We assume that this sentiment in teachers' language, fuelled by their enthusiasm, also contributes to important facets of teaching quality, such as the provision of support and autonomy and the creation of caring relationships (Fauth et al., 2019; Gaspard & Lauermann, 2021).

## TEACHER ENTHUSIASM AND SENTIMENT IN THEIR TALK

### Conceptualising teacher enthusiasm

Teacher enthusiasm is a complex construct, and different authors have emphasised different aspects about it. In the present contribution, we follow the tradition of viewing teacher enthusiasm as an affective‐motivational teacher characteristic involving passion for the process of teaching and its content, and outward expressiveness to convey one's energy and activation to the students. This sets our conceptualisation apart from other works that equated teacher enthusiasm with teaching enjoyment, hence focusing on the internal emotional experience of the teacher (e.g., Cui et al., 2020; Lazarides et al., 2021), or older works that considered teacher enthusiasm only in terms of displayed teacher behaviours (Collins, 1978; Murray, 1983). We concur with Taxer and Frenzel (2018), who argued that teachers' internal experiences and their outward teaching behaviours might not always align and that purely outwardly displayed energetic behaviour in the absence of internally experienced enjoyment reflects dissonance and inauthenticity. In the present study, we explicitly addressed *authentic enthusiasm*, defined as the *coupling of teaching enjoyment with outward positive expressiveness* (Keller et al., 2016).

### Why should teacher enthusiasm manifest itself in teacher talk?

There are multiple conceivable behavioural channels through which teacher enthusiasm can manifest itself in overt teacher behaviours, including verbal and non‐verbal features. A majority of existing research focused on non‐verbal behavioural aspects, such as movement towards students or facial, vocal, and gestural expressions (see Babad, 2007, for a review). Verbal features involve specific verbal content; for example, teachers' use of humour in the class, self‐disclosure, complimenting students' performance, use of inclusive pronouns, or addressing students by name (see Witt et al., 2004, for a review). Clearly, language is a primary means for people to express their thoughts and emotions and to communicate values to one another (e.g., Maia & Santos, 2018; Rosenberg & Hirschberg, 2009). Accordingly, it is plausible to assume that teachers' enthusiasm is reflected in the language they use during teaching. However, whether or not teachers' enthusiasm is linked with the semantically transmitted overall *emotional valence* of their language, or more specifically, whether it is a higher quantity of positively connoted words used and/or a lower quantity of negatively connoted words, is yet unknown. To the best of our knowledge, no study to date seems to have explored the link between teacher enthusiasm and the emotional tone of teachers' language.

### Using sentiment analysis to measure teacher talk

Sentiment analysis is a suitable tool for quantifying the emotional tone, opinion, or subjectivity of a text (Liu, 2020; Pang & Lee, 2008). Sentiment is quantified in terms of valence (positive vs. negative; e.g., great vs. terrible) and intensity (e.g., considering that the word “great” is stronger than “good”; Liu, 2020; Taboada et al., 2011). Hence, sentiment analysis can be used to detect emotions or attitudes of the producer of a text or a speaker.

The idea that texts or spoken utterances can carry emotional valence to differing degrees dates back several decades (e.g., Osgood et al., 1957). Today's sentiment analysis is a branch of natural language processing (NLP) rooted in computational linguistics. There are lexicon‐based and machine‐learning sentiment analysis approaches. The lexicon‐based approach involves large lists of words of a given language (lexica) that are tagged in terms of their so‐called prior polarity, i.e., their emotional valence and intensity, independent of the context they are used in (Liu, 2020; Wang et al., 2019). Employing modern computational power, every single word in a given text or speech transcript is then systematically compared with these lexica; each word is assigned its lexicon‐implied sentiment value, so that a total parameter is obtained that represents the text's overall sentiment.

Machine‐learning approaches, in turn, use supervised learning to train algorithms on the basis of labelled instances of texts or sentences, which can then be applied to new texts or transcripts to estimate their sentiment. While such classifiers perform very well in the domain that they were trained on, their performance drops precipitously when the same classifier is used in a different domain (Taboada et al., 2011). Given that there is no established machine‐learning model to classify the sentiment in teaching contexts, we opted for a lexicon‐based approach, using SentiWS, a publicly available German sentiment lexicon (Remus et al., 2010, version 2.0 from 2019; see https://wortschatz.uni‐leipzig.de/de/download).

So far, the primary purposes for sentiment analysis in educational research were to evaluate students' feedback and to improve learning and teaching practices (Zhou & Ye, 2020). To the best of our knowledge, the present study is the first to employ sentiment analysis on extended transcripts of secondary school teachers’ in‐class talk to determine the emotional tone they express during teaching. In doing so, we deliberately chose to restrict the context, as the subject domain might play a role in the typical vocabulary used by teachers and how they use certain words. We contextualised the present study in the subject domain of mathematics. Mathematics is a core subject, and mathematical skills are essential for a large range of professions (National Research Council, 2013); hence, school success in mathematics is highly important in most societies. At the same time, mathematics is a subject considered as hard and unattractive by many students (Schoenfeld, 2022), which is why scientifically supported insight into how mathematics teachers can enthuse them about the subject is of great significance (Boaler, 2022).

## HABITUAL AND IN‐SITU ENTHUSIASM

Our goal of exploring links between teachers' enthusiasm and the emotional tone of their language had an important methodological implication: To capture teachers' language, we had to “zoom in” to the situational level of a specific lesson, which we videotaped to obtain the audio transcripts for the sentiment analysis. It therefore seemed reasonable to also assess the teachers' in‐situ enthusiasm with respect to this particular lesson—in addition to the classic way of assessing teacher enthusiasm as a personal habit or trait (Keller et al., 2016). This allowed us to explore the question of whether such habitual and in‐situ enthusiasm ratings are linked.

It is a long‐standing theoretical proposition that a specific human action results from an interaction between personality characteristics and situational demands (for more recent accounts, see e.g., Fleeson & Jayawickreme, 2015; Lewin, 1936). Strikingly, empirical evidence regarding the links between habitual teacher characteristics and their in‐situ classroom behaviour is sparse. Cui et al. (2017) reported that student ratings of their teachers' habitual enthusiasm were systematically positively linked with students' in‐situ ratings of task value and negatively linked with student boredom in a specific lesson. Further, Feng and colleagues (Feng et al., 2021, 2023) showed that teachers' habitual intrinsic orientation to teach—of which they conceptualised enthusiasm to be a key facet—was weakly positively linked with in‐situ observer ratings of teaching quality.

## THE PRESENT STUDY

The key goal of the present contribution was to examine the way how teachers' enthusiasm translates into their classroom behaviour, specifically the way they speak. To this end, we obtained teacher self‐reported and student‐perceived ratings of habitual teaching enthusiasm and analysed the teachers' verbally expressed sentiment as extracted from the transcribed audio tracks of video recordings of one full mathematics lesson of these teachers. We additionally recorded the teacher self‐reported and student‐perceived in‐situ enthusiasm as assessed directly after the videotaped lesson. First, we systematically explored whether habitual teacher enthusiasm (teacher‐reported or student‐perceived) was linked with teachers' and students' in‐situ ratings of teacher enthusiasm in the given lessons. Second and most importantly, we investigated whether both habitual and in‐situ teacher enthusiasm were linked with the emotional tone of teachers' language spoken during teaching. Our study hypotheses were the following.

Hypothesis 1 *(“personality–situation realisation hypothesis”)*: There are systematic links between teacher enthusiasm conceptualised as a habitual and as an in‐situ state phenomenon.

Given that we assessed teacher enthusiasm through teacher self‐report and student ratings, we specifically tested whether

H1.1 teachers' self‐reports of their habitual enthusiasm were linked with their self‐reports of enthusiasm in one specific lesson.

H1.2 student ratings of their teachers' habitual enthusiasm were linked with their ratings of that teacher's enthusiasm in one specific lesson.

Hypothesis 2 *(“enthusiasm–language manifestation hypothesis”):* More enthusiastic teachers express more positive sentiment in the natural language they use in the classroom.

Specifically, we tested whether

H2.1 teachers' self‐reports of their habitual and in‐situ enthusiasm were (a) positively linked with the average sentiment in their talk, (b) positively linked with the positive valence of their talk, and (c) negatively linked with the negative valence of their talk.

H2.2 student ratings of their teachers' habitual and in‐situ enthusiasm were (a) positively linked with the average sentiment in their talk, (b) positively linked with the positive valence of their talk, and (c) negatively linked with the negative valence of their talk.

Further, to get deeper insights into the language teachers use in their classroom talk reflecting their enthusiasm, we pursued additional exploratory analyses. Specifically, we explored which were the most frequently used words tagged with a positive sentiment by SentiWS and which were the words with the strongest positive valence used. Additionally, we identified words used by the teachers that seemed to be particularly relevant in conveying enthusiasm. We deemed those qualitative analyses as fruitful to learn about which positively connoted words actually are used in real classrooms, in order to inform potential future interventions targeted at teacher enthusiasm trainings. Further, those in‐depth analyses promise to pave the road to future research refining LLM algorithms for quantifying instruction‐specific positive sentiment.

## METHOD

### Study design and participants

The data reported herein stem from the longitudinal school field study FEEL (see also Burić & Frenzel, 2023; Schwartze et al., 2024) conducted at eleven secondary schools in Bavaria, Germany. This project was designed to track mathematics teachers and one of their classes for one school year, with four self‐report survey measurement points across the school year, plus one video assessment administered past midterm. The video recordings were complemented by short post‐lesson self‐report diaries filled out by teachers and students. The project was approved by the Institutional Ethics Review Board of the first author's institution and had ministerial consent by the Bavarian state Ministry of Education.

Initially, the project involved 93 mathematics teacher‐class tandems. Participation in the video and corresponding lesson diary assessment was voluntary for the teachers. We obtained informed consent from 21 teachers. Next, we additionally obtained parents' and students' informed consent that videotaping may happen in these classes, which was denied in two classes.

The resulting final sample for the present analyses thus consisted of *N* = 19 secondary school mathematics teachers (*n* = 9/10 identifying as male/female) and their *N* = 393 students (*n* = 204/143 female/male, *n* = 46 not specified) from fifth to tenth grade of all three school types of the Bavarian tripartite secondary school system (*n*_teachers_ = 12/2/5; *n*_students_ = 284/41/67 from academic/intermediate/vocational track; *n*_teachers_ = 5/2/8/1/2/1; *n*_students_ = 77/39/173/31/46/27 from grades 5/6/7/8/9/10). On average, 20.68 students participated per class.

### Measures

#### Teacher enthusiasm

**FIGURE 1:**
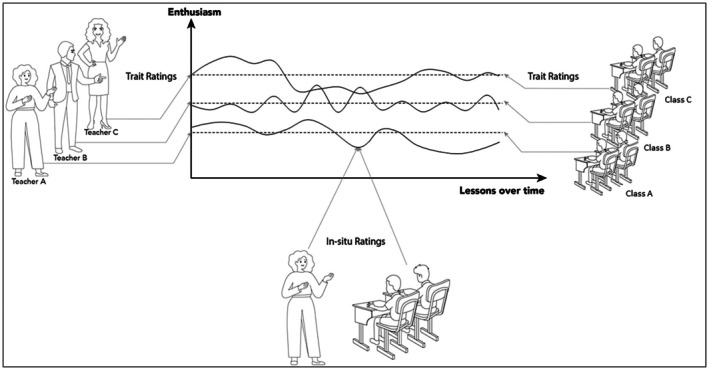
Visualisation of the trait‐ and in‐situ approaches to measuring teacher enthusiasm, as rated through the teachers' self‐reports and their classes' average perceptions. Depiction of enthusiasm fluctuation smoothed and between‐teacher differences exaggerated for demonstration. Figure available at https://figshare.com/account/articles/26312407 under a Creative Commons Attribution Licence (https://creativecommons.org/licenses/by/4.0/), which permits unrestricted use, distribution, and reproduction in any medium, provided the original work is properly cited.

We included teachers' self‐reports and students' perceptions. Item formulations corresponded with our definition of authentic enthusiasm as the coupling of internal experiences with outward positive expressiveness (see Table 1 for a full list). All items were rated on five‐point Likert scales from 1 [*strongly disagree*] to 5 [*strongly agree*]. Figure 1 visualises the data structure and approaches to variable operationalizations as realised in the present study, depicting lesson‐to‐lesson fluctuations as well as person‐typicality of teachers' enthusiasm.

**TABLE 1:** Overview of items used in the present study [alongside their original German item versions used in this study].

Teacher trait enthusiasm: teacher self‐report	I enjoy teaching these students. [In dieser Klasse macht mir Unterrichten Freude.] 2 I often have reason to be happy while I teach these students. [Während des Unterrichtens in dieser Klasse habe ich oft Grund, mich zu freuen.] 3 I teach these students with enthusiasm. [In dieser Klasse unterrichte ich mit Begeisterung.] 4 In this class, I try to get the students enthusiastic about the subject matter. [In dieser Klasse versuche ich im Unterricht, die Schülerinnen und Schüler von den Unterrichtsinhalten zu begeistern.] 5 In this class, I try to convey the fascination of my subject to the students. [In dieser Klasse versuche ich, den Schülerinnen und Schülern die Faszination an meinem Unterrichtsfach zu vermitteln.]
Teacher trait enthusiasm: student perceptions	My math teacher… [Meine Mathelehrkraft…] seems to enjoy teaching very much. [scheint großen Spaß am Unterrichten zu haben.] teaches with enthusiasm. [unterrichtet mit Begeisterung.] tries to get us excited about mathematics. [versucht im Unterricht, uns vom Fach Mathematik zu begeistern.] teaches with humour. [unterrichtet mit Humour.] emphasises what he/she says with gestures and facial expression. [unterstreicht mit ihrer Gestik und Mimik, was sie sagt.]
Teacher in‐situ enthusiasm: teacher self‐report	In the past lesson… [In dieser Schulstunde …] I enjoyed teaching. [hat mir das Unterrichten Spaß gemacht.] I tried to enthuse the students about class. [habe ich versucht, die Schülerinnen und Schüler vom Unterricht zu begeistern.]
Teacher in‐situ enthusiasm: student perceptions	In the past lesson… [In dieser Schulstunde…] my teacher enjoyed teaching. [hat meiner Lehrkraft das Unterrichten Spaß gemacht.] my teacher taught with enthusiasm. [hat meine Lehrkraft mit Begeisterung unterrichtet.]

*Note*: All items were answered on a scale from 1 [*strongly disagree*] to 5 [*strongly agree*].

##### The teacher self‐reported trait enthusiasm

Teachers' habitual self‐reported enthusiasm teaching the target class was measured using five items (see Table 1), tapping both the inner experiential and the outward‐expressive facet of enthusiasm (Frenzel et al., 2016; Taxer & Frenzel, 2018). It was assessed in the second‐wave surveys of the longitudinal study, so the teachers and their classes had sufficient in‐class experience together by the time they provided those ratings. The scale demonstrated good internal consistency with a coefficient omega of .87.

##### Student‐rated trait enthusiasm

For students, the perceived teacher enthusiasm scale from Taxer and Frenzel (2018) was used, which consists of five items (see Table 1). It was assessed in the second‐wave surveys of the study, too, and showed good internal consistency (omega: .76) on the individual student level. More importantly, class‐average ratings of teachers' enthusiasm differed considerably across classes, as demonstrated by a high intra‐class correlation (ICC1) of .28 and a corresponding high ICC(2) of .86 for the class‐average teacher trait enthusiasm (Luedtke et al., 2009).

##### Teacher‐ and student‐rated in‐situ lesson enthusiasm

In‐situ enthusiasm was assessed directly after the videotaped lesson through two items by teachers and students each (see Table 1). The two items were substantially correlated from the perspective of the students (omega for the 2‐item scale on the student level was .72) and the corresponding reliability of the class‐average ratings was good (ICC(1) = .12; ICC(2) = .64). For teachers, the correlation was lower (with an omega of .17 for the 2‐item scale). Nevertheless, as we sought to capture both the inner experiential and outward expression components of the teachers' in‐situ enthusiasm, we built a mean composite score across the two items for both students and teachers.

#### Teacher sentiment

##### Transcription from classroom video recordings

Teachers were videotaped using small action cameras (GoPro© Hero 4) which were equipped with two mono microphones for audio recordings at 48 kHz at the front and the back of the camera. Teachers were told that they could move freely during the session, that they would be videotaped once they were within the camera's field of view, and audiotaped continually.

We used the software Sonix (https://sonix.ai) for an initial automated transcription of the audio files obtained in the videotaped lessons. Next, all teacher utterances from the AI‐supported transcripts were manually cleaned by a native German student assistant. Cleaning involved (a) corrections regarding speaker misspecification (teacher vs. students) and (b) corrections of semantic content of the teacher utterances (e.g., due to dialect).

##### Sentiment analysis procedure

We used the SentiWS lexicon (Remus et al., 2010) for our sentiment parameter extraction. In the assembly of the SentiWS, the General Inquirer (Stone et al., 1966), a co‐occurrence analysis (Dunning, 1993), and the German Collocation Dictionary (Quasthoff, 2010) were used as sources. The weighting was carried out using the Pointwise Mutual Information Method (Church & Hanks, 1990), a common method (Taboada et al., 2011) that uses a specific word in the dictionary and tests its distance regarding a predefined set of particularly positive or negative words (see Remus et al., 2010, and https://wortschatz.uni‐leipzig.de/de/download for more details). SentiWS contains not only adjectives and adverbs but also nouns and verbs that carry sentiment. These sentiment scores are standardised to range between −1 and +1, indicating valence in varying absolute values (e.g., +0.3716/+0.7299 for “good”/“perfect”; −.1593/−.3042 for “stupid”/“terrible”). The current version of SentiWS contains a total of around 16,000 positive and 18,000 negative word forms (including base forms and all their German inflections). Thus, it can be applied directly to original transcripts without any pre‐processing (e.g., reducing words to their word stems).

For our sentiment analysis, we used the full transcripts of all teacher utterances in the lesson. First, the transcripts were divided according to words, so that each word of a teacher was listed individually in the data set. Subsequently, each word was compared against the words within SentiWS. Those words that were present in the lexicon were then scored with the corresponding sentiment score. From this, we obtained three sentiment indexes for each teacher: (1) an overall sentiment index by averaging all sentiment scores of the individual words within each teacher's transcript, (2) a positive sentiment index by averaging only the scores of the positively valenced words in each transcript, and (3) a negative sentiment index by averaging the scores of the negatively valenced words in each transcript.

### Statistical analyses

Analyses for this study were conducted using R (R Core Team, 2023) and the R packages tidyverse (Wickham et al., 2019), tidytext (Silge & Robinson, 2016), and quanteda (Benoit et al., 2018). All anonymised data and code are available on OSF (https://osf.io/fwvbs/?view_only=d715bc285f324b37ba32a181abd07486). Missing data on the individual item level was handled by obtaining conditional means, resulting in no missing data on the enthusiasm scales, both for the teacher and for the student datasets. In order to test our hypotheses, we ran a series of Kendall's tau correlation analyses using the R package ppcor (Kim, 2015). We chose a non‐parametric analysis approach due to the small sample size and because the teacher enthusiasm ratings, specifically the in‐situ ratings, which were means across only two items on a five‐point Likert scale, could not be considered interval scaled. The parameters thus represent the degree to which the two variables of interest have a monotonic association.

In addressing Hypothesis 1, proposing systematic links across teachers' habitual and in‐situ realised enthusiasm, we obtained correlations between teacher enthusiasm as measured using trait‐based habitual scales, on the one hand, and in‐situ situational scales, on the other; for both teacher self‐reported and student‐rated perspectives. In addressing Hypothesis 2, we obtained partial correlations across the teachers' sentiment scores and teacher‐ as well as student‐rated habitual and in‐situ enthusiasm, controlling for the word count of the teacher transcripts. We judged correlations as statistically significant with *p*‐values below .05 and classified correlation sizes according to Gignac and Szodorai (2016); considering correlations of 0.10, 0.20, and 0.30 as relatively small, typical, and relatively large.

## RESULTS

### Preliminary analyses

Table 2 displays the means and standard deviations of the teacher self‐reported and student‐rated enthusiasm scales as well as the sentiment indexes. Teachers' own endorsement of the items, but also average student ratings, were relatively high on the 5‐point scale. The total number of words in the transcripts of all teachers in our sample was 48,496 (*M*_transcript length_ = 2,552 words; Min = 1,368 words, Max = 3,833 words). The large majority of those words (95.1%) did not carry any sentiment. This is an expectable proportion, as SentiWS contains predominantly adjectives, verbs, and nouns, but no function words such as prepositions or pronouns, which do not carry any sentiment but make up a large proportion of natural language. In turn, of the remaining 2,363 words that were tagged with a sentiment below or above zero by SentiWS, 81.8% were positive, and 18.2% were negative. Of note, while a clear majority of the words were positively connoted, the average positive valence of those words was weaker (*M* = 0.15, Min = 0.04, Max = 0.73) than the average negative valence of the relatively less frequently used negatively connoted words (*M* = −0.20, Min = −0.95, Max = −0.04). Overall, this resulted in a slightly positive total average sentiment expressed by the teachers in our sample (*M* = 0.09, Min = 0.01, Max = 0.18). Finally, relative to their means, the standard deviations for all three sentiment indeces per teacher were also comparably large in size, implying that there was considerable inter‐individual variability in the sentiment as expressed in the teachers' spoken language in class.

**TABLE 2:** Descriptive statistics of study variables.

Construct	*M*	*SD*
Trait enthusiasm		
Teacher self‐report	4.00	0.66
Class‐aggregated perception	3.74	0.47
In‐situ enthusiasm		
Teacher self‐report	4.08	0.56
Class‐aggregated perception	3.94	0.40
Sentiment		
Overall mean	0.09	0.05
Positive mean	0.15	0.06
Negative mean	−0.20	0.05

*Notes*: *N* = 19. Possible range for the enthusiasm ratings was 1–5.

### Links between trait and in‐situ ratings of teacher enthusiasm (“Personality–Situation Realisation Hypothesis”)

Table 3 displays the correlations across the habitual and the in‐situ teacher enthusiasm scores, as rated by teachers themselves as well as their students (see Table S1 for a full bivariate correlation matrix). These results lent partial support to our personality‐situation realisation hypothesis (H1). Teachers' self‐reported habitual enthusiasm, as reported in the trait questionnaire, administered multiple weeks before the videotaped lesson, correlated as highly as .49 with their self‐reported enthusiasm as reported directly after the videotaped lesson. For the class‐average student ratings, the correlation across trait‐based and in‐situ ratings was .22, hence of typical size (Gignac & Szodorai, 2016). However, this parameter did not reach statistical significance, likely due to the relatively small sample size.

**TABLE 3:** Kendall's tau coefficients across habitual and in‐situ ratings of teacher enthusiasm.

		In‐situ enthusiasm					
		Teacher self‐report			Class mean perception		
		Kendall's tau	*p*	CI	Kendall's tau	*p*	CI
Habitual Enthusiasm	Teacher self‐report	.49	.009	[0.20; 0.70]	.37	.033	[0.05; 0.62]
	Class mean perception	−.01	.942	[−0.33; 0.31]	.22	.195	[−0.11; 0.50]

*Note*: *N* = 19.

Above and beyond these habitual/in‐situ correlational links within the teacher‐ and class‐based ratings, results across the perspectives were mixed. While teachers and their classes did not converge in their judgements regarding how enthusiastic a teacher generally is, they agreed substantially in their perceptions of how enthusiastic the teacher was in the one specific lesson we videotaped for the present study.

### Links between teacher enthusiasm and verbal sentiment (“Enthusiasm–Language Manifestation Hypothesis”)

Table 4 shows the partial correlations between teacher‐ and student‐reported enthusiasm and verbally expressed sentiment, controlling for word count (see Table 3, first column). In view of the small sample size, we also report post‐hoc power for each obtained parameter. In line with our hypothesis (H2), there were significant, large positive correlations between teachers' self‐reported enthusiasm and their overall verbal sentiment as expressed during the videotaped lesson; this pertained both to their self‐reports of habitual teaching enthusiasm and to their post‐lesson diary‐based in situ enthusiasm reports. Likewise, the correlation between the classes' aggregated ratings of their teachers' in‐situ enthusiasm and teacher sentiment score was typical in size (Kendall's tau = .21), but it did not reach statistical significance. Not in line with our hypothesis, there were no consistent links between student‐rated teacher trait enthusiasm and teacher verbal sentiment.

**TABLE 4:** Kendall's tau coefficients across teacher enthusiasm and sentiment indices.

	Sentiment (overall mean)			Post‐hoc power	Positive sentiment			Post‐hoc power	Negative sentiment			Post‐hoc power
	Kendall's tau	*p*	CI		Kendall's tau	*p*	CI		Kendall's tau	*p*	CI	
Trait Enthusiasm												
Teacher self‐reports	.38	.029	[0.06; 0.62]	.50	.44	.011	[0.14; 0.67]	.62	.00	.980	[−0.33; 0.32]	.05
Class‐aggregated perceptions	−.14	.429	[−0.44; 0.20]	.14	−.18	.286	[−0.48; 0.15]	.18	.10	.573	[−0.23; 0.41]	.11
In‐situ Enthusiasm												
Teacher self‐reports	.52	.002	[0.24; 0.72]	.77	.58	<.001	[0.31; 0.76]	.86	.06	.726	[−0.27; 0.38]	.08
Class‐aggregated perceptions	.21	.233	[−0.13; 0.50]	.22	.23	.183	[−0.10; 0.51]	.25	.03	.865	[−0.30; 0.35]	.06

*Note*: *N* = 19. Partial correlations controlling for word count are shown. Post‐hoc power was obtained through GPower with the following settings: test family: exact; statistical test: correlations; type of power analysis: compute achieved power‐given alpha, sample size, and effect size; input parameters: tail(s) = One, correlation ρ H1 = [observed r], α err prob. = 0.05, total sample size = 19, correlation ρ H0 = 0.

Next, we inspected the teacher enthusiasm/sentiment links as broken down by positive and negative sentiment indices (see Table 3, second and third column). The result pattern clearly showed that teacher enthusiasm was substantially linked with the positive sentiment index, but correlations with the negative sentiment index were consistently zero.^1^

### Insights into teachers' emotional language in class: Exploratory analyses

Given that it was predominantly the positive sentiment that was systematically linked with teachers' enthusiasm, we focused our exploratory analyses on the emotionally positively connoted words. Specifically, we inspected (a) which were the most frequently used words tagged with a positive sentiment by SentiWS, (b) which were the words with the strongest positive valence used, and (c) which words were used by the teachers that seemed particularly relevant in conveying enthusiasm.

As can be seen in Table 5, the most frequently used positive words were not particularly strongly positively connoted (e.g., good), while the words with particularly high SentiWS‐assigned valences were used considerably less frequently. One exception was the strongly positively connoted word “super,” which was used with considerable frequency. Digging deeper into the use frequency of this word for each teacher revealed that “super” was used by 12 out of the 19 teachers in our sample, and among those, there were three who used it 6 or 7 times per lesson, while all others used it only once (*n* = 3), twice (*n* = 3), or three/four times (*n* = 1 each). This implies that such specific word usage also depends on individual style.

**TABLE 5:** Top‐ranking positively valenced words used by the teachers in our sample.

Positively valenced words with highest frequency of use				Frequencies of words with highest SentiWS‐assigned valence			
German	English translation	SentiWS‐assigned sentiment score	Frequency	German	English translation	SentiWS‐assigned sentiment score	Frequency
gut	good	.3716	226	perfekt	perfect	.7299	13
genau	exact	.0040	201	wunderbar	wonderful	.7234	7
richtig	correct	.0040	134	spannend	exciting	.7165	6
einfach	easy	.0040	118	wunderschön	beautiful	.7048	4
groß	large	.3694	77	hervorragend	excellent	.5891	5
hoch	high	.0040	58	toll	great	.5066	2
schön	nice	.0081	43	super	super	.5012	37
klar	clear	.0040	43	genießen	savour	.4983	2

*Notes*: *N* = 19 teachers; *N* = 1932 words denoted as positively valenced by SentiWS used in total. For the German terms, any inflection of words was included in the word count (e.g., the count for “gut” included usage of “gute” or “guter”).

To identify words that seemed to be particularly relevant in conveying enthusiasm, five independent raters (blind to the word frequencies) went through the entire list of words used by the teachers (excluding the most frequent positive words and the most positively valenced words already addressed in our prior analyses, reported in Table 5) and marked their “top 12” enthusiasm‐conveying words. Table 6 shows the use frequencies of all words named by at least two out of the five raters. Important insights from these findings were that teachers explicitly used the German word “begeistert” (which translates into enthused/enthusiastic) very rarely—in fact, only two teachers used it exactly once each in the videotaped lesson. The explicit reference to “enjoyment” was also made seldomly; one teacher used it twice, another one once, in expressing their own enjoyment; once it was used in the context of a student's enjoyment, and once in the context of “we”—hence meaning the teacher and the class together. The words “interest” or “interesting” were used with a comparably higher frequency, even if not as frequently as one might assume, expecting that teachers would want to emphatically express the value of a lesson's subject content. However, it is worth noting that the word “important”, which also conveys value, was used with considerable frequency; in fact, it rated among the most‐used positively connoted words in total, alongside “nice” and “clear” (see Tables 5 and 6).

**TABLE 6:** Frequencies of enthusiasm‐conveying words.

German	English translation	SentiWS‐assigned sentiment score	Frequency
begeistert	enthused/enthusiastic	.4324	2
interessant/interessieren	interesting/interested	.2488	18
freuen	enjoy	.2198	5
großartig	great	.4606	1
lieb	kind	.1131	1
mögen	like/be fond of	.3450	22
besonders	special	.0040	7
cool	cool	.0040	4
erstaunlich	astonishing	.0040	1
gefallen	like/please	.2578	2
lachen	laugh	.0135	1
positiv	positive	.0040	2
witzig	funny	.4463	3
wichtig	important	.3822	43

*Note*: For the German terms, any inflection of words were included in the word count (e.g., the count for “freuen” included usage of “freue” or “freut”).

## DISCUSSION

Despite substantial empirical evidence on the nature of positive effects of teacher enthusiasm, theoretically well‐founded explanations and empirical evidence on the specific behavioural manifestations and resulting effects of teacher enthusiasm are rare (Keller et al., 2016). Existing evidence heavily draws on non‐verbal facets of teacher enthusiasm; verbal facets considered so far pertained to specific semantic content (e.g., self‐disclosure) but not its emotional valence. We addressed this gap by exploring links between teachers' enthusiasm and the sentiment of their language.

To quantify teachers' sentiment, we submitted transcripts of audio tracks of video recordings of full mathematics lessons to sentiment analysis using a lexicon‐based approach (Remus et al., 2010). Teacher enthusiasm was assessed through classical trait‐based measures of teachers' enthusiasm as shown habitually in the classroom, and through post‐lesson diaries for in‐situ ratings of the teachers' enthusiasm as shown specifically during the videotaped lessons. Both teacher self‐reports and student perceptions were obtained. As such, this study was unique in combining three data sources: teacher self‐reports, student ratings, and the sentiment scores as obtained from the teachers' transcribed utterances in class.

In line with our “personality‐situation realisation hypothesis”, habitual enthusiasm was positively correlated with in‐situ enthusiasm as rated by teachers and students. This supports the idea that when teachers describe themselves as typically high in teaching enthusiasm, this indeed manifests itself in corresponding high levels of felt and expressed enthusiasm also in a one‐shot, single situational instance several months later. However, the trait/in‐situ correlation was considerably smaller for the class‐aggregated student ratings. This may imply that students provide less reliable and consistent ratings of their teachers' enthusiasm than teachers. A reason for this could be that students do not give their teachers' mental states and strategic instructional intentions so much thought, as for them, it is an other‐ rather than a self‐rating (see also Vazire, 2010, self‐other knowledge asymmetry model).

Further and more importantly, in line with our “enthusiasm‐language manifestation hypothesis,” we found consistent positive links between teachers' self‐reported enthusiasm and their verbal sentiment as expressed during the lesson. This pertained both to self‐reports of their habitual teaching enthusiasm, and to their post‐lesson diary‐based in‐situ enthusiasm reports. Importantly, this enthusiasm/sentiment link was clearly driven by the positive sentiment index, which showed even stronger correlations with teacher enthusiasm than the overall score. In contrast, teacher enthusiasm was completely unrelated to the valence strength of negatively connoted words by the teachers. In conclusion, teacher enthusiasm specifically seems to drive the valence intensity of positively connoted words in the classroom, while it does not at the same time dampen the negative valence of teachers' negatively connoted word use. Notably, the positive and negative sentiment scores tended to be positively correlated (Pearson's *r* = .17, *p* = .48). Hence, the sentiment teachers express in the classroom is not a unidimensional phenomenon—teachers who tend to use more strongly positively valenced words do not use less strongly negatively valenced words. On the contrary: If anything, we observed a trend for “emotional intensity” in teachers' spoken language, with certain teachers using more strongly emotionally connoted words of either valence.

These teacher‐reported enthusiasm/sentiment correlations were large in size, underlining that the teachers' language as used in the classroom is clearly associated with the degree to which they refer to themselves as being a generally enthusiastic mathematics teacher and with the degree to which they reported being enthusiastic in a specific lesson. The correlation for the in‐situ rating tended to be even higher than the correlation for the trait rating, which seems reasonable as these two scores were obtained at the same level of situational granularity, namely the videotaped lesson.

Considering students as informants for teachers' enthusiasm, however, the sentiment as expressed in the teachers' language in class was not systematically linked with students' class‐aggregated perceptions of their teachers' habitual enthusiasm and was comparably weakly positively associated with student‐rated in‐situ enthusiasm. Again, this could be due to the fact that students are the less reliable source of inquiry for assessing teacher enthusiasm (Fauth et al., 2020; Vazire, 2010). Alternatively, this finding may also imply that for some teachers, the sentiment in their language reflects their attempts to “send” messages about the value and enjoyability of mathematics, which, however, are not properly “received” by their students (see also Hall et al., 2019, for deliberations about discrepancies between sending and receiving nonverbal messages). For example, students may assign a not particularly positive meaning to some of their teachers' utterances, even if they are explicitly positive in valence (to the point that they may perceive a statement like “this is a really great task” as sarcastic). In other words, even with identical levels of emotional sentiment in their language, some teachers may be more successful than others in verbalising their enthusiasm towards their students. Accordingly, the fact that students may not be as sensitive about picking up their teachers' sentiment in interpreting their teaching enthusiasm habit might be one of the points at which the “signal gets lost” during the chain from affective‐motivational teacher characteristics, via teaching quality, to student outcomes (Bardach & Klassen, 2021). Alternatively, it may also be that nonverbal facets (tone of voice, facial expression, posture/movement) override verbal facets of enthusiasm on the receiving student end (Witt et al., 2004). Lastly, a more refined index of teacher emotional sentiment (see below) could reveal closer alignment also with student‐perceived teacher enthusiasm.

Overall, we propose that our findings provide important new evidence that it is the positive sentiment in teachers' language in their day‐to‐day interactions with students that sets highly enthusiastic teachers apart from their less enthusiastic colleagues. The present study also provided important first insights into the scientific workflow for realising sentiment analysis through automated, AI‐supported audio transcription coupled with manual cleaning. Future studies expanding on our work could explore the links between teachers' sentiment and facets of teaching quality that have been proposed to be linked with teacher enthusiasm, such as the provision of social support and autonomy, or the creation of caring relationships (Fauth et al., 2019; Gaspard & Lauermann, 2021).

## Limitations and implications for future research

There were a few design properties of the present study that compromised its validity and posed certain limits to the generalisation of its findings. Those include the relatively small teacher sample size, the fact that the study context was restricted to mathematics classrooms, and the fact that the study was conducted in only one cultural context. The key finding of the present study—that teacher self‐reported enthusiasm is positively linked with the positive sentiment of their in‐class talk—was substantiated by a post‐hoc power of .86 for the in‐situ ratings and .66 for the trait ratings; hence it is unlikely that those were mere chance findings. Nevertheless, it will be essential to replicate the present findings using larger, more representative samples from various contexts.

The specific focus of the present study on the domain of mathematics potentially affected our results. For example, the term “negative”, which obviously carries a negative sentiment in an abstract sense, can be used in a rather neutral, purely mathematical sense of absolute value of numbers.^2^ Further, the words “exact,” “easy,” or “large” may have had a higher use propensity in mathematics than they would have by teachers in other subject domains. An intriguing question that arises is whether a word such as “large” potentially carries a lower valence and hence, conveys less enthusiasm when used in a mathematical sense compared to different domain contexts, such as philosophy or literature. For example, speaking of “a large question” in such contexts might be particularly positively connoted and explicitly convey enthusiasm. Likewise, in German one would use the term “large” when speaking of a “great poet”, using large to denote exceptionality, which carries a decidedly positive connotation.

Further, research has shown effects of culture on emotion display rules in the classroom (Hagenauer et al., 2016), thus the degree to which the verbal and nonverbal expression of teacher enthusiasm is deemed appropriate may well vary across cultures. Nevertheless, we speculate that the basic psychological mechanism addressed in the present study, namely that teacher enthusiasm drives teachers' use of positively connoted words in their classroom talk, applies universally–that is, across subject domains, cultures and languages.

Clearly, a major challenge for the present study was finding an adequate German emotion lexicon for the sentiment analysis. Compared to English sentiment lexica and models, German ones currently still perform less well (Fehle et al., 2021). We chose to select Remus et al.'s (2010) emotion lexicon SentiWS v2.0 because it is the broadest lexicon we know of and was one of the best‐performing lexica according to a comparison conducted by Fehle et al. (2021). For the assembly of sentiment lexica, sources are often retrieved from quite narrow domain contexts. For example, the PotTS (Sidarenka, 2016) or the GermEval‐2017 (Wojatzki et al., 2017) used very specific–and clearly non‐school‐related contexts to develop their lexica, such as review portals, news (Wojatzki et al., 2017), or social media posts (Sidarenka, 2016); hence we doubted their transferability to the context of teaching.

Further, a downside that comes with a purely lexicon‐based approach to sentiment analysis is that word classification does not consider the context in which it is used, for example, if a word is preceded by a negation (e.g., not interesting) or an intensifier (e.g., very interesting). In principle, natural language processing technology has developed algorithms to account for this; the best‐known being the Bidirectional Encoder Representations from Transformers (BERT) model, which accounts for the text both before and after a word. For German sentiment analysis, the only BERT‐trained model (Guhr et al., 2020) was trained on hotel or movie reviews; hence its applicability to the school context was highly doubtful. Further, Frenky and Betey (2023) proffered an emotion generation model rooted in data mining of teacher‐student course interactions in an educational context, yet in an extremely narrow field, namely online physical education. We propose that our results showing substantial correlative links between the sentiment score as obtained using SentiWS and teacher enthusiasm speak for themselves, given that the purely lexicon‐based sentiment index it produced was a rather “blunt sword” in quantifying the teachers' emotional tone. We demonstrated that the positive valence strength of teachers' emotionally connoted words, irrespective of the semantic context they used them in, was systematically and substantially linked with teachers' self‐rated enthusiasm.

Future research involving teacher sentiment could explore opportunities for training teaching‐specific sentiment models by using student and teacher ratings of their in‐class behaviour and experiences as criteria. In so doing, lexicon‐based and machine‐learning approaches to sentiment analysis could be combined to result in optimal, pretrained sentiment algorithms that detect teacher speech characterising teacher enthusiasm. The body of words used by the teachers in the present study, alongside the teacher‐ and student‐rated enthusiasm, could be a starting point for such enthusiasm‐focused sentiment algorithm development. Yet, teaching enthusiasm is but one example here—sentiment algorithms could also be trained to predict specific facets of teaching quality, detecting teacher utterances conveying autonomy provision, student rapport, or structure and clarity. This future research may also reveal the optimal granularity of contextual specificity for the corresponding algorithms—for example, whether different algorithms would need to be designed for mathematics versus other subject domains.

Finally, the present study was limited in addressing one single verbal aspect of teacher enthusiasm, even though this construct clearly also comprises non‐verbal aspects (Babad, 2007). As such, words may not only shift in meaning depending on the subject context they are used in, but also depending on other cues, including nonverbal ones, sent along by the speakers. Hence, a promising path for future research exploring affective facets of instructional processes also may lie in multi‐modal sentiment and emotion recognition (e.g., Viegas & Alikhani, 2021; Wang et al., 2019). Clearly, it is not only *what* teachers say (i.e., the emotional sentiment of their spoken language in class) but also *how* they say it. For example, emotions can be reflected in speech rate and pitch variation (Breitenstein et al., 2001), and facial, gestural, and spatial behaviours of teachers complement the semantic messages they send to their students.

## Conclusions and implications for teaching practice

The present study's insights into which enthusiasm‐carrying words are typically used by real teachers in day‐to‐day classroom teaching could be applied in virtual avatar‐supported learning to optimise authenticity. Additionally, they can be used for teacher training to boost teachers' enthusiasm. It has been shown that a positive school climate, teacher efficacy, and leadership are linked with teacher enthusiasm (Öngel & Tabancalı, 2022; Zhang & Ye, 2023), and that enthusiastic behaviours are generally trainable (e.g., Bettencourt et al., 1983). Our findings could be used for specific teacher trainings, supporting them to use a more positive verbal language. While up‐regulating one's non‐verbal positive expressiveness may come with emotional costs to those with a more introverted personality (cf. literature on teacher emotional labour, e.g., Wang et al., 2019), a change in the language may come easier for such individuals. The propositional nature of language, coupled with the complexity afforded by vocabulary and syntax, makes verbal behaviour more precise, useful, and flexible than non‐verbal behaviour (Patterson et al., 2023) and hence subject to intentional behavioural change. Generally, we do propose that such trainings bear promise, as prior research has shown that instructors' positive emotions expressed in class are reciprocated by their learners, which can contribute to the teachers' well‐being during teaching (Frenzel et al., 2024).

## AUTHOR CONTRIBUTIONS

**Anne C. Frenzel:** Conceptualization; investigation; funding acquisition; writing – original draft; methodology; visualization; formal analysis; resources; project administration; data curation. **Hannah Kleen:** Writing – original draft; writing – review and editing; validation; formal analysis; data curation; methodology. **Anton K. G. Marx:** Validation; writing – review and editing; formal analysis; data curation; investigation. **David F. Sachs:** Validation; writing – review and editing; data curation. **Franziska Baier‐Mosch:** Writing – review and editing. **Mareike Kunter:** Writing – review and editing; resources; supervision; conceptualization.

## Supporting information


Table S1.

## Data Availability

The data that support the findings of this study are openly available in OSF at https://osf.io/fwvbs/?view_only=d715bc285f324b37ba32a181abd07486

## Footnotes

^1^
Given that it can also be argued that teacher enthusiasm should be conceptually distinguished into an experiential subfacet (teaching enjoyment) and a behavioural subfacet (expressed enthusiasm; Taxer & Frenzel, 2018), we reran our analyses to obtain separate correlations of these subfacets with the sentiment indices. The results mirrored those reported for the composite score. For teachers, there were large and consistent links across both teaching enjoyment and expressed enthusiasm, on the one hand, and their sentiment, on the other. For students, there were positive links only with student perceptions of in‐situ teacher joy and expressed enthusiasm, but not with their trait ratings. In sum, these results showed that the separate correlation parameters for the sentiment indices and those potential two subfacets of teaching enthusiasm were highly similar.

^2^
While we were lucky that this did in fact not compromise our present findings, as the word “negative” was used only one single time by one of the teachers in our sample; here indeed in the context of a negative (as opposed to positive) value of a number.
