# Supplementary material for: Is it in their words? Teachers' enthusiasm and their natural language in class–A sentiment analysis approach
Source: Br J Educ Psychol. 2025 Jan 31;95(Suppl 1):S32–51. doi: 10.1111/bjep.12734 (PMC12427161; doi:10.1111/bjep.12734)
Supplement: Supplementary file 1 — Table S1. [file BJEP-95-S32-s001.pdf]

Table S1: Correlation matrix across all study variables

| | Trait Enthusiasm (Teacher Self-Report) | Trait Enthusiasm (Student Perceptions) | In-Situ Enthusiasm (Teacher Self-Report) | In-Situ Enthusiasm (Student Perceptions) | Sentiment (overall mean) | Positive Sentiment | Negative Sentiment | Wordcount |
|---|---|---|---|---|---|---|---|---|
| Trait Enthusiasm (Teacher Self-Report) | 1.00 | -.01 | .49** | .37* | .39* | .45** | .02 | -.10 |
| Trait Enthusiasm (Student Perceptions) | | 1.00 | -.01 | .22 | -.11 | -.16 | .11 | -.10 |
| In-Situ Enthusiasm (Teacher Self-Report) | | | 1.00 | .45* | .51** | .56** | .05 | .06 |
| In-Situ Enthusiasm (Student Perceptions) | | | | 1.00 | .20 | .22 | .02 | .02 |
| Sentiment (overall mean) | | | | | 1.00 | .77** | .36* | -.16 |
| Positive Sentiment | | | | | | 1.00 | .22 | -.16 |
| Negative Sentiment | | | | | | | 1.00 | -.22 |
| Wordcount | | | | | | | | 1.00 |

*Notes*. $N = 19$. All parameters shown are Kendall's tau coefficients.
